# Metaphorical construction of the belt and road initiative in German media: a transnational study based on critical metaphor analysis

**DOI:** 10.3389/fpsyg.2026.1828657

**Published:** 2026-04-23

**Authors:** Haiying Qiu

**Affiliations:** Department of Foreign Language, Shanghai Customs University, Shanghai, China

**Keywords:** belt and road initiative, conceptual metaphor, critical metaphor analysis, cross-cultural communication, German media discourse

## Abstract

**Introduction:**

Media representations of the Belt and Road Initiative (BRI) play a significant role in shaping public understanding of China’s flagship transnational initiative. Despite growing scholarly interest in BRI discourse, the metaphorical mechanisms underlying German media coverage remain underexplored.

**Methods:**

Drawing on Conceptual Metaphor Theory (CMT) as its theoretical foundation and employing Critical Metaphor Analysis (CMA) as its analytical approach, this study adopts a corpus-based mixed-methods design to analyze 1,247 articles totaling 856,432 words from four major German mainstream media outlets spanning 2013 to 2024.

**Results:**

The analysis identifies 2,847 metaphorical expressions across five dominant conceptual frames: JOURNEY (31.3%), WAR (24.0%), BUILDING (18.3%), ORGANISM (15.4%), and GAME (11.0%). Temporal analysis reveals a marked shift toward confrontational framing, with WAR metaphors increasing from 18.6% to 31.2% over the observation period. Cross-national comparison positions German media between Anglo-American confrontational discourse and Chinese cooperative narratives.

**Discussion:**

The findings demonstrate that metaphorical framing operates as a cognitively structured and ideologically consequential mechanism in transnational political communication, with systematic variation across political orientations and temporal phases reflecting broader shifts in Sino-German geopolitical relations.

## 1 Introduction

The Belt and Road Initiative (BRI) has grown from being a conceptual framework in 2013 to one of the most expansive initiatives representing global coordination across the realms of global politics today. As this particular proposal transforms global politics along economic avenues, the role of the media in shaping public discourse around the BRI amid China’s increasing global engagement cannot be overstated. Media representations of the BRI within their respective ideological frameworks have produced significant regional differences in coverage: Australian news media narratives experienced dramatic transitions between 2013 and 2021 (Jiang, 2024), while Indian and Pakistani media adopted starkly contrasting stances reflecting regional political tensions (Chu et al., 2025). Studies from Kazakhstan (Slamgazhy et al., 2024) and Austria (Li and Yang, 2025) further illustrate how countries with different geopolitical positions construct divergent media images of the initiative, demonstrating the highly context-dependent nature of BRI discourse construction.

European media discourse on the BRI offers a particularly illuminating case. As a major economic and political bloc with complex ties to China, Europe has undergone a notable shift in its perception of both the BRI and China more broadly (Breslin and Mattlin, 2025). This transformation has been catalyzed by multiple factors, including China’s role in reshaping EU trade defense mechanisms (Comerma, 2025) and the evolving geopolitical considerations that continue to filter their perception of China through a security prism. In this larger European environment, the role of the German news media assumes a position of strategic importance. As the continental power of the European Union and a strong exponent of the policy of engagement between the EU and China, the news discourse in Germany not only shapes public discourse at home but also sets the tone of continental sentiment. The importance of Germany in the context of the BRI is fourfold. First, Germany is China’s largest trade partner in the EU and its most influential economic power. As such, the Sino-German relationship plays a key role in shaping the overall policy directions of the EU towards China. Second, Germany is an important hub in the BRI’s infrastructure connectivity plan, with the Duisburg terminal being the EU’s primary hub for China-Europe Railway Connections, accounting for a significant portion of the total China-Europe Railway Freight traffic. Third, the geopolitical position of Germany, balancing Atlanticism with economic cooperation with China, creates a particularly ambivalent discursive field, one likely to yield more nuanced and complex metaphorical framing than national contexts that are more clearly aligned with or opposed to China. Fourth, due to Germany’s prominence within the EU and the broader European political process, its media discourse exerts a particularly significant influence on the European media landscape as a whole. The role of news media in shaping public perception regarding the BRI has been attested to in a number of contexts (Oo and Dai, 2024), while there can be detected in the American media a pattern of negative sentiment with typological attributes (Malik and Hou, 2025). Yet despite this strategic importance, German-language media coverage of the BRI remains underexplored in academic research.

Existing research on media representations of the BRI has predominantly employed content analysis or framing analysis. For instance, Jiang (2024) analyzed shifts in Australian media narratives of the BRI using framing analysis, while Chu et al. (2025) applied comparative content analysis to Indo-Pakistani media coverage. Similarly, Slamgazhy et al. (2024) examined BRI coverage in Kazakhstan through an agenda-setting framework. However, the role of metaphor as a fundamental cognitive device remains underexplored. Unlike explicit framing strategies, metaphors operate at a deeper cognitive level, structuring how audiences comprehend and evaluate complex policy initiatives. Cross-national research has found large variations of discourse of BRI across nations (García-Herrero and Schindowski, 2023; Wei and Hu, 2024). However, the above-mentioned research has not investigated the metaphorical construction systematically as a prime methodological approach, especially from a European linguistic environment. This is particularly relevant because metaphors serve as the primary means through which the public comprehends and evaluates abstract political and economic initiatives.

To address these gaps, this study conducts a Critical Metaphor Analysis of BRI representations in mainstream German media from 2013 to 2024. Drawing on CMT as its theoretical foundation and employing CMA as its analytical approach, the study pursues three objectives: (1) identifying the dominant metaphorical frames used to conceptualize the BRI in German media discourse; (2) tracing the temporal evolution of these metaphorical patterns across the observation period; and (3) uncovering the ideological orientations embedded in the selection and deployment of specific metaphorical constructions.

## 2 Literature review and theoretical framework

### 2.1 Critical metaphor analysis: from rhetoric to ideology

Metaphor research in discourse analysis has also experienced a paradigm shift from conceptualizing metaphors as secondary rhetorical flourishes to seeing them as basic cognitive and ideological tools. Critical Metaphor Analysis (CMA), which combines the findings of cognitive linguistics and the critical approach of discourse analysis, has proved to be an advanced approach in unmasking the role of metaphorical discourse in naturalizing specific worldviews and particular political aims. Recent theoretical developments have extended CMA by incorporating cross-linguistic comparison and diachronic analysis (Liu et al., 2024), enabling researchers to trace metaphorical variation across linguocultural groups and over time in response to shifting sociopolitical contexts. The integration of corpus methods has substantially enhanced the rigor and scalability of metaphor research. Meta-reviews through corpus research confirm the ability of computers to systematically track metaphorical patterns in large text databases, while also highlighting the challenges that arise when manual analyses are conducted at the micro-level (Abdul Malik et al., 2022). The empirical applications of the concept of corpus-based CMA have found to be valuable in the demonstration of the strategic usage of metaphors in political contexts. An example of the study of the usage of the metaphor of war in COVID-19 coverage of the Pakistani media illustrates the usage of metaphorical frames in the legitimation of policy actions (Rana et al., 2024). All this research points to the importance of metaphor analysis being aware of the sociocultural and political context in which certain metaphors achieve prominence.

The efficacy of CMA in critically analyzing news and political discourse has been successfully demonstrated in various studies. For instance, Lu and Yu (2024) employed a critical corpus-assisted approach to analyze how metaphors contribute to the construction of crisis discourses surrounding COVID-19 vaccines in Chinese and American news media, revealing how metaphors systematically reflect ideological differences between the two contexts. In a related study, Lu (2026) utilized a critical corpus-assisted discourse analysis approach to investigate how strategic discourses in US governmental discourse on Russia and Ukraine shifted across the Obama, Trump, and Biden administrations, demonstrating how metaphors and other forms of rhetorical devices respond to changing geopolitical dynamics. Collectively, these studies highlight the efficacy of critical corpus-assisted approaches in analyzing news and political discourse, serving as a methodological precedent for this study’s application of CMA to German BRI discourse. In addition, Zeng and Ahrens (2023) successfully demonstrated the efficacy of combining critical interpretive analysis with CMA in their analysis of WAR metaphors in Hong Kong public discourse, showing that quantitative patterns can be systematically linked to real-world politics.

### 2.2 Conceptual metaphor theory: cognitive foundations and extensions

Conceptual Metaphor Theory has dramatically altered the understanding of the role of metaphor in the human mind since its conceptualization by Lakoff and Johnson. Conceptual Metaphor Theory argues that metaphor is not a peripheral linguistic phenomenon but a fundamental cognitive mechanism through which abstract concepts are understood in terms of concrete experiential domains. Conceptual Metaphor Theory has been especially enlightening when analyzing political discourse because metaphors determine the way in which the public conceptualizes political realities and judges political proposals.

Some recent applications of Conceptual Metaphor Theory (CMT) bring out its ability to provide an analytical lens through which the political circumstances of the day can be studied. Analysis of conceptual metaphors in Donald Trump’s 2024 election campaign discourse reveals how political actors deploy specific metaphorical models, such as the NATION metaphor, to frame political realities (Giang and Hiep, 2025). In the same manner, the study of the usage of words in the presidential debates of 2024 reveals the ways through which the opposing political groups employ distinctive linguistic strategies to express their ideological stances (Wicke and Bolognesi, 2025). These studies illustrate that metaphor choice is far from arbitrary but reflects and reinforces fundamental political worldviews.

The current trends in research concerning CMT are shifting towards a more dynamic and contextualized approach to metaphorical thinking. The conceptual metaphor approach of Kövecses stresses the point that understanding metaphors does not remain constant but fluctuates due to the factors of context and purpose of communication (Kövecses, 2020). This model of theoretical evolution illustrates particular significance in the context of research regarding cross-cultural political communication because it suggests the possibility of metaphorically formulating the same initiative—the example used being the BRI—even in profoundly different manners according to the national media context.

CMT is particularly well-suited for such an analysis, as conceptual metaphors operate outside conscious awareness, rendering them particularly effective ideological tools. Moreover, unlike other argumentation strategies that can be deployed intentionally, metaphorical framing structures readers’ understanding of complex policy initiatives in terms of experiential domains, thereby constraining the inferences readers can draw (Lu and Yu, 2024). This cognitive dimension positions CMT as an indispensable supplement to the critical orientation of CMA. The dual nature of metaphors, as both cognitive and ideological tools, has been theoretically established (Demjén and Semino, 2020). Moreover, integrated approaches combining metaphor analysis with framing theory have empirically demonstrated their capacity to reveal the cognitive and ideological functions of metaphors in applied studies such as health communication (Semino et al., 2018). However, studies that systematically combine both dimensions using corpora are scarce, justifying the adoption of this integrative framework in this study.

### 2.3 Discourse construction of the belt and road initiative

The Belt and Road Initiative has been the flagship of the current Chinese foreign policy and the international economic agenda of the PRC and has attracted vast amounts of research regarding the discourse construction of the policy. Studies about the discourse of the BRI problem point to the fact that the meaning of the policy can be found neither in a simple message of the policy itself nor within discourse itself but also in the interaction of discourse and the realities of the policy. Hu (2024) employs an assemblage approach to explain how BRI discourse operates through multiple layers of interaction across various scales of discourse practice, promoting standardization within the global political economy through its application to diverse geographical contexts.

To understand the discourse of BRI, it is important to contextualize it within the framework of the strategic narratives of the People’s Republic of China. Scholarly work on the strategic narratives of the People’s Republic of China regarding the reform of global governance under the leadership of Xi Jinping has found innovations in discourse to construct the image of China’s role and aspirations abroad (Yang, 2021). These strategic narratives play various roles: internal legitimation of the government’s foreign policy positioning and the contestation of existing narrative frameworks dominated by the West. However, the reception and subsequent reinterpretation of the narratives remain widely different at the national level through the existing media systems’ own political perspectives concerning the geopolitical positioning of the Chinese government.

The dynamics of the discourse surrounding the BRI in the online environment also introduce various complexities. The study of the dynamics of the BRI community on the Twitter platform during the COVID-19 pandemic showed the existence of clear polarization of the discourse, where the groups of users supporting and opposing the BRI retreated into the “echo chambers” of their own discourse (Man et al., 2025). Moreover, studies on China’s COVID-19 diplomacy have shown how European media framed China’s pandemic assistance, acknowledging its “humanitarian” dimension while also questioning the “strategic” rationale behind it (Qi et al., 2022), a pattern that mirrors metaphorical tensions observed in the BRI discourse. Finally, studies on China’s strategic narratives in vaccine diplomacy have shown how the media in different national contexts adapt these narratives to local frameworks and pre-existing perceptions of China (Stojiljković and Mihelj, 2025). This further supports the idea that the metaphorical dimension is not merely a reflection of the situation but rather constitutes the actual intersection between media cultures and geopolitical stances. By the same token, the study of the Chinese government’s news coverage of global happenings, say the story tactics of the Global Times about the conflict in Ukraine (Tran Sautedé et al., 2025), illustrates how state-affiliated media construct interpretive frameworks that align with national strategic interests. Research on Chinese diplomatic discourse, facilitated by the construction of comprehensive corpora of Foreign Ministry press conferences (Mochtak and Turcsanyi, 2021), enables systematic tracking of how official narratives evolve in response to shifting international circumstances.

The intersection of media discourse and policy formation is particularly pertinent in the field of BRI studies. In the context of the EU’s geoeconomic turn, the study of frames and issue linkage in media discourse indicates how discursive framing shapes policy responses to the changing global economic order (Christou and Damro, 2024). This underscores the potential importance of metaphorical constructions of the BRI in European media, particularly regarding their impact on public opinion and the formulation of European policy responses. Further evidence comes from the study of conceptual metaphors in Chinese ecological discourse, which points to the cross-cultural importance of path-oriented conceptualizations as a cognitive mechanism underpinning development initiatives (Chunxiang, 2024). Similarly, research on the reframing of China in US trade policy discourse through context-deictic space models indicates a parallel process of repositioning China as a strategic competitor rather than an economic partner (Hu and Li, 2025), a process that is replicated in the context of German media in the present study.

Research on BRI discourse across various national media settings has identified consistent differences in both metaphorical and ideological strategies. Comparative research on Chinese and American news discourse, for instance, has identified how metaphorical and rhetorical strategies differ in response to China’s global activities (Malik and Hou, 2025; Wei and Hu, 2024). Similarly, research on Russian media discourse has identified that media outlets with different political affiliations in the same national context employ distinct metaphorical strategies to represent the BRI (Kuteleva and Vasiliev, 2021), indicating political ideology as a key shaping factor. Corpus-assisted research on Chinese official diplomatic discourse has identified recurring ideological themes characterized by cooperative and development-oriented metaphorical strategies (Liu et al., 2023). These findings provide a point of reference for positioning critical metaphorical strategies in German media discourse.

### 2.4 Integrating conceptual metaphor theory and critical metaphor analysis

While both CMT and CMA offer valuable analytical tools, their combined potential has rarely been explored. CMA focuses on the importance of metaphor in ideology and the naturalization of power relations, but in doing so, it may pay insufficient attention to cognitive mechanisms of metaphor’s persuasiveness. Conversely, CMT has provided rich insights into these cognitive mechanisms but has underexplored how metaphorical choices relate to ideology. This study aims to introduce a framework that integrates the strength of both approaches: on one hand, CMT’s core insight that metaphorical understanding is based on cognitive mappings from concrete source domains to abstract target domains; on the other, CMA’s fundamental assumption that metaphorical choices always carry ideological weight, naturalizing worldviews while obscuring potential alternatives.

The framework has four interrelated components. Corpus-based identification identifies metaphorical expressions through the computational power of semantic analysis software linked with manual validation. Conceptual categorization classifies metaphorical expressions according to the type of source concept (JOURNEY, WAR, BUILDING, ORGANISM, and GAME), in line with the principles of Conceptual Metaphor Theory. The mapping component examines the particular conceptual ties and relationships entailed in the interaction between the two concepts and identifies the components of the BRI construct that are foregrounded and those which are backgrounded through metaphor. The critical phase interprets the metaphorical mappings within the construct of the BRI through various ideological complex constructions across different media sources reflecting divergent political ideals.

Applying this framework to the coverage of the BRI in the German media allows the research to systematically examine the ways in which metaphorical framing contributes to the German public’s understanding of the Chinese proposal. By isolating the predominant metaphorical configurations and their development over time and unearthing the underlying ideological meaning of the metaphorical narratives being used in the discourse, the research sheds light on the ways in which the German media negotiate and perhaps contest the ideological narratives employed in the Chinese proposal regarding the BRI.

The integrative approach adopted in the current study also builds on existing traditions in the analysis of political rhetoric. Charteris-Black (2018) framework, which analyzes political speeches through the combined lens of rhetoric, discourse, and metaphor, offers a precedent for examining the relationship between metaphor and other forms of argumentation in the context of political rhetoric. This rhetorical dimension is particularly relevant to the current study’s focus on how German media develop evaluative positions in relation to the BRI through systematic metaphorical selection that simultaneously reflect and reinforce geopolitical positionings.

## 3 Data and methods

### 3.1 Data

The corpus consists of news articles from four representative German mainstream media sources spanning from 2013 to 2024, covering the entire period of the BRI’s existence. The media sources were selected to represent a diverse range of political orientations and target audiences.

Der Spiegel represents Germany’s leading news magazine with investigative journalism tradition and center-left orientation. Die Zeit serves as the country’s most prominent weekly newspaper, known for in-depth analysis and intellectual discourse. Frankfurter Allgemeine Zeitung (FAZ) constitutes Germany’s newspaper of record with conservative-liberal positioning and strong influence in business and political circles. Süddeutsche Zeitung represents Germany’s largest subscription newspaper with center-left editorial stance and significant readership in southern Germany.

Data collection employed systematic keyword-based searches using the terms “Belt and Road Initiative,” “Neue Seidenstraße” (New Silk Road), “Seidenstraßeninitiative” (Silk Road Initiative), and “BRI” in combination with “China.” Articles explicitly discussing the BRI or its associated projects were included, while brief mentions without substantive content were excluded. This resulted in a corpus of 1,247 articles totaling 856,432 words.

The corpus spans from the formal announcement of the BRI in September 2013 to the end of December 2024, covering all stages of BRI discourse from early introduction to mature development, and is therefore well-suited for analyzing the evolution of metaphorical structuring patterns across these stages. The distribution of articles over time reflects growing media interest following major BRI-related events: article counts were relatively low between 2013 and 2016, increasing significantly from 2017 onwards after the first Belt and Road Forum. The conservative FAZ and the center-left Der Spiegel produced the highest number of articles. By ensuring the corpus is representative of different political leanings, this research captures the range of positions evident in German public discourse regarding this flagship international policy initiative. The corpus composition is presented in Table 1.

**Table 1:** Corpus composition by media source.

Media source	Political orientation	Article count	Word count	Percentage
Der Spiegel	Center-left	342	238,156	27.4%
Die Zeit	Liberal	286	201,847	22.9%
Frankfurter Allgemeine Zeitung	Conservative	358	247,892	28.7%
Süddeutsche Zeitung	Center-left	261	168,537	20.9%
Total	–	1,247	856,432	100%

Percentage is calculated based on article count relative to the total corpus (*N* = 1,247 articles).

Articles were retrieved through the LexisNexis Academic database and the respective media outlets’ digital archives, which provide authorized access to full-text newspaper and magazine content for academic research purposes. For articles not available through database access, texts were obtained from publicly accessible portions of the outlets’ websites. Text extraction was performed using the Python libraries BeautifulSoup (for HTML parsing) and PyMuPDF (for PDF text extraction) to convert the collected materials into standardized plain text format. Metadata including publication date, media outlet, article type (news report, editorial, feature, or commentary), and author information were systematically recorded for each text. The corpus was annotated using the UAM CorpusTool for systematic coding and retrieval. Linguistic preprocessing involved tokenization, lemmatization, and part-of-speech tagging using the spaCy library with German language models, facilitating subsequent computational metaphor identification and pattern analysis.

### 3.2 Methods

This study employs corpus-based Critical Metaphor Analysis (CMA), drawing on Conceptual Metaphor Theory (CMT) as its theoretical foundation. Following Tognini-Bonelli (2001) distinction, this study adopts a corpus-based approach, in which pre-established theoretical categories from CMT guide the analysis of corpus data, rather than a corpus-driven approach where categories emerge exclusively from the data itself. A mixed-methods design combines computational corpus linguistics methodologies with critical discourse analysis, enabling the systematic identification of metaphorical patterns alongside their ideological interpretation.

Metaphor identification followed a systematic protocol combining computational assistance with manual verification using the Metaphor Identification Procedure VU University Amsterdam (MIPVU; Steen et al., 2010). Automated candidate detection was performed using the UCREL Semantic Analysis System (USAS) (Rayson et al., 2004), which coded the corpus for semantic field information; cases where contextual semantic information differed from the default semantic field were flagged as potential metaphor candidates. Each candidate was then manually verified through the MIPVU steps: (a) reading the full textual context, (b) establishing the basic meaning of the lexical unit, (c) comparing the contextual meaning with the basic meaning, and (d) confirming the metaphorical function where the contextual meaning differed from but could be inferred by the basic meaning.

Each confirmed metaphor was categorized into source domain categories—JOURNEY, WAR, BUILDING, ORGANISM, and GAME—in accordance with CMT principles, and further coded for evaluative orientation (positive, neutral, or negative) and ideological function. Critical analysis then examined the ways in which these metaphorical choices articulate specific representations of the BRI, with particular attention to the ideological work accomplished through patterns of metaphor selection across different media outlets.

### 3.3 Research procedures

**Figure 1:**
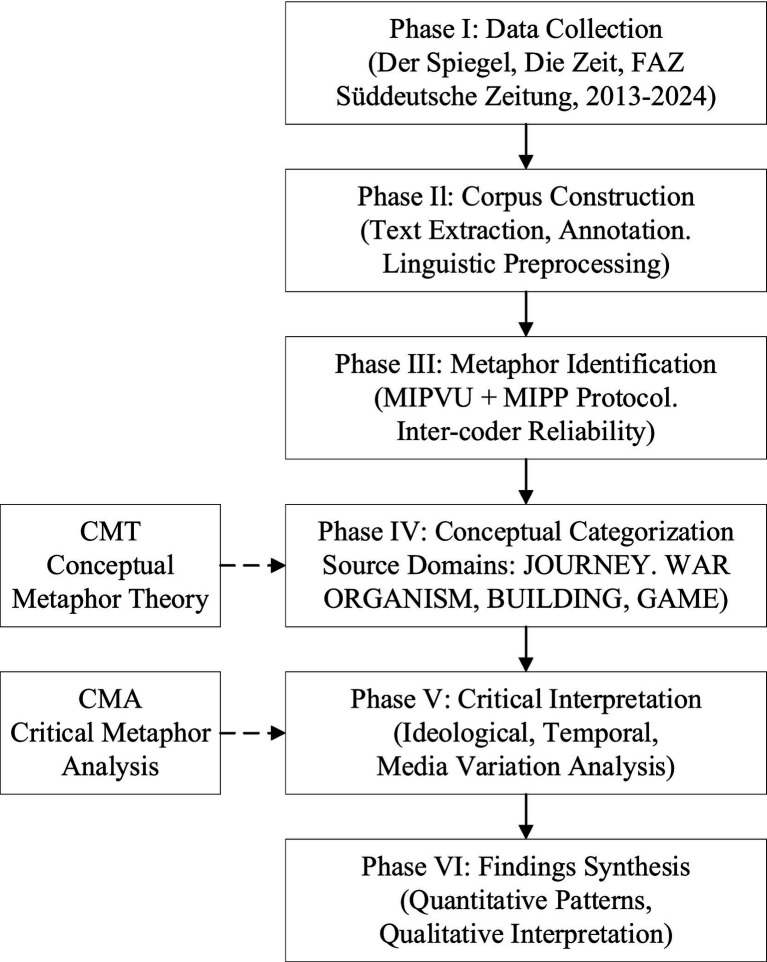
Research framework for corpus-based critical metaphor analysis of BRI discourse.

The analytical procedure follows a multi-step protocol, as presented in Figure 1. The research first employed systematic data collection from the four representative German news sources described in Section 3.1, followed by corpus construction and linguistic annotation. Metaphor candidates were subsequently identified through automated computational processing using USAS, and each candidate was manually verified using MIPVU. Confirmed metaphors were then systematically catalogued according to source domain categories and coded for evaluative orientation and ideological function.

The critical phase examined how the identified metaphorical frameworks construct specific representations of the BRI, scrutinizing the ideological mappings embedded in the metaphorical constructions across different media sources reflecting divergent political orientations. The final stage synthesized the quantitative distributional findings with qualitative critical interpretation to provide an integrated account of the metaphorical framing of the BRI in German mainstream media discourse.

## 4 Findings

### 4.1 Dominant conceptual metaphor frames in German media

The overall study of the metaphorical expressions in the corpus shows that there are five conceptual metaphor frames through which the German media conceptualize the BRI. As presented in Table 2, the findings from the identification of the metaphorical expressions in the corpus showed that there are a total of 2,847 metaphorical expressions of which JOURNEY metaphors took the highest number followed by metaphors of WAR, BUILDING, ORGANISM, and GAME, respectively. This pattern of numbers showed that the discourse of the German media on the BRI showed a preference of conceptualization through the spatial-directional conceptual metaphor frames.

**Table 2:** Distribution of conceptual metaphor types in German BRI discourse.

Metaphor type	Source domain	Token count	Type count	Percentage
JOURNEY	Path, Road, Destination	892	156	31.3%
WAR	Battle, Strategy, Territory	684	132	24.0%
BUILDING	Construction, Foundation, Structure	521	98	18.3%
ORGANISM	Growth, Body, Life cycle	438	87	15.4%
GAME	Competition, Rules, Players	312	64	11.0%
Total	–	2,847	537	100%

“Type Count” refers to the number of distinct lexical types (lemmas) per category.

The dominance of JOURNEY metaphors fits ideally within the spirit of the name of the scheme itself as far as the invocations of historical trade routes are concerned, but the study reveals that the conceptual mapping of German media involves not only geographical interlinkages but also the ideas of progress, direction, and movement. The WAR metaphors come next as the second dominant conceptual relation that reveals the natural drift of the German press coverage of the BRI to be situated within the context of competitive geopolitical prism of the scheme entailing moves, territorial occupation, and antagonistic roles. The BUILDING metaphors take the next position according to which the scheme can be conceptualized as a constructive endeavor involving foundations and structural development. ORGANISM metaphors conceptualize the BRI as a living entity with processes of growth, expansion, and potential decline, while GAME metaphors frame the initiative within competitive dynamics involving strategic calculation and rule-governed interaction.

**Figure 2:**
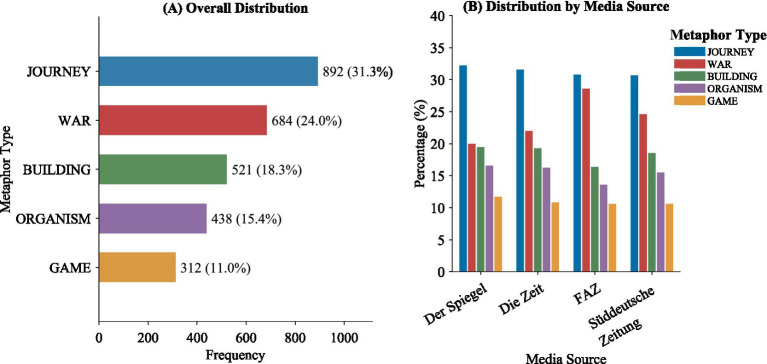
Distribution of conceptual metaphors in German BRI discourse.

As evident in Figure 2, distribution reveals high levels of variation when different media houses are considered. FAZ, which has a leaning towards the right side of the political spectrum, presents a remarkably high usage of metaphors of WAR when compared to centrally left media houses. However, the usage of JOURNEY and BUILDING metaphors in Der Spiegel and Süddeutsche Zeitung is marked. These distributional patterns suggest that political orientation is associated with distinct preferences in metaphor usage within BRI discourse.

The Type-Token Ratio (TTR) provides additional insights into the lexical diversity and cognitive productivity of each metaphor type. GAME metaphors have the highest TTR (0.224), which indicates that despite their relatively low frequency, they draw on a diverse range of lexical items. This lexical diversity suggests that GAME metaphors have not yet become fully conventionalized in German BRI discourse: their metaphoricity remains more perceptible to readers, which means that their ideological work—constructing Sino-European relations as a rule-governed, zero-sum strategic competition—is more explicit and requires more active interpretive participation from the audience. ORGANISM metaphors similarly exhibit high lexical diversity (TTR = 0.197), reflecting the rich biological and ecological vocabulary available within this source domain. This diversity indicates that the cognitive mapping of the BRI onto organic growth processes is realized through varied and creative lexical choices, suggesting an ideological function oriented toward naturalizing the BRI’s expansion as an autonomous, quasi-biological process that operates independently of human political agency. BUILDING metaphors occupy a moderate position (TTR = 0.190), suggesting that although their constructional vocabulary is relatively standardized around terms such as “foundation,” “structure,” and “framework,” sufficient lexical variation exists to allow contextually nuanced critiques of the BRI’s feasibility and stability. JOURNEY metaphors, the most frequent category overall, display a similarly moderate TTR (0.183), reflecting a balance between highly conventionalized path-related vocabulary and contextually varied directional expressions: this combination enables the JOURNEY frame to function both as a seemingly neutral descriptive frame and, when deployed with evaluative directional terms, as a vehicle for ideological positioning regarding China’s navigational dominance in the BRI. In contrast, WAR metaphors display the lowest TTR (0.175), which suggests that this type of metaphor is highly conventionalized and that a limited range of words is used repeatedly. This low TTR also reveals that this type of metaphor is highly entrenched in media discourse about the BRI in Germany, to the point where its metaphorical nature is no longer consciously perceived by readers—functioning instead as a conventionalized or ‘dead’ metaphor in the cognitive-linguistic sense (Lakoff and Johnson, 1980). This entrenchment carries significant ideological consequences: precisely because WAR metaphors are not recognized as metaphors, they naturalize a zero-sum, adversarial framing of the BRI as an invisible cognitive default, making alternative interpretive frames—such as mutual economic benefit or cooperative development—cognitively less accessible.

### 4.2 Critical analysis of representative metaphorical expressions

To exemplify the ideological role of the identified metaphorical patterns, this section presents representative corpus examples and discusses their critical relevance to power relations, identity construction, and ideological naturalization.

WAR metaphors hold particularly prominent ideological significance in the construction of the geopolitical threat narratives. For example, the expression “China erobert mit der Seidenstraße strategische Positionen in Europa” (China conquers strategic positions in Europe through the Silk Road, FAZ, 2021) employs the verb erobert (conquer) to equate military-style territorial acquisition with economic investment. This metaphor naturalizes a zero-sum understanding of BRI infrastructure development. Moreover, it constructs China as the aggressor, thereby legitimizing defensive policy actions, such as the EU’s Foreign Direct Investment Screening Regulation, by presupposing the invasion dynamic in the relationship between China and the European market. The term strategische Positionen (strategic positions) further militarizes the discourse, framing the acquisition of port facilities or rail terminals as covert military-strategic maneuver. Similarly, the expression “Die neue Seidenstraße ist Pekings schärfste Waffe im Kampf um globale Einfluss” (The New Silk Road is Beijing’s sharpest weapon in the battle for global influence, Der Spiegel, 2020) metaphorically casts the BRI as a weapon (Waffe) in the geopolitical arena, which is characterized as a battle (Kampf). The expression constructs the BRI not as an economic development program but as a geopolitical weapon, foreclosing interpretations centered on mutual economic benefit, while positioning European nations as targets requiring defensive action.

JOURNEY metaphors, although seemingly collaborative, reveal patterns of uneven power relations under closer critical analysis. For instance, “Peking bestimmt die Route der neuen Seidenstraße – Europa muss aufpassen, nicht vom Weg abzukommen” (Beijing determines the route of the New Silk Road – Europe must be careful not to lose its way, Die Zeit, 2019) places Beijing in a position of sole navigation, bestimmt (determining) the course, whereas Europe is relegated to a passive role, forced to follow and risk losing its way. Rather than affirming China’s “shared journey” narrative, this metaphor reifies BRI as a one-way journey, with the imperative “aufpassen” (“be careful”) implicitly casting Europe as vulnerable.

BUILDING metaphors carry out significant evaluative functions inasmuch as they construct the BRI as a structurally vulnerable enterprise. The phrase “Das Fundament der neuen Seidenstraße zeigt bereits Risse” (The foundation of the New Silk Road is already showing cracks, FAZ, 2022) implies the BRI’s infrastructure development initiatives are founded on structurally unstable economic or political grounds. Such framing essentializes BRI skepticism in terms of feasibility, thereby legitimizing European hesitation to participate based on perceived structural unsustainability.

ORGANISM metaphors present the expansion of the BRI as organic yet potentially uncontrolled growth. For example, the words “Die Seidenstraße wächst wie ein Organismus, der sich in alle Richtungen ausbreitet” (The Silk Road grows like an organism that spreads in all directions, Süddeutsche Zeitung, 2020) suggest invasive, uncontrolled proliferation, presenting the BRI as a dehumanized entity operating beyond the domain of political decision-making.

Finally, GAME metaphors situate the Sino-European relations in the context of competitive strategic interaction. In the following quote, the metaphor of the chess game is used: “Im geopolitischen Schachspiel der Seidenstraße hat Europa bisher nur reagiert” (In the geopolitical chess game of the Silk Road, Europe has so far only reacted, Die Zeit, 2021). This quote suggests elements of strategy, self-interest, and zero-sum game outcomes. Most significantly, however, it positions “Europe” as a reactive actor in the game of geopolitical strategies with China in the context of the BRI.

### 4.3 Temporal evolution of metaphorical patterns

The findings from the diachronic study of metaphorical framing patterns over the 11-year period of observation show that there has been a marked change in the conceptualization of the Belt and Road Initiative from the German media’s perspective. The study has been able to ascertain that there are three marked stages of development marked by preference for various conceptual metaphors – Introduction Phase (2013–2016), the Expansion Phase (2017–2019), and the Contestation Phase (2020–2024), as presented in Table 3.

**Table 3:** Temporal distribution of conceptual metaphors across three phases.

Metaphor type	Introduction phase (2013–2016)	Expansion phase (2017–2019)	Contestation phase (2020–2024)
JOURNEY	42.3% (186)	32.8% (298)	24.5% (367)
WAR	18.6% (82)	22.4% (204)	31.2% (467)
BUILDING	15.2% (67)	21.6% (196)	16.8% (252)
ORGANISM	14.5% (64)	14.9% (135)	16.2% (243)
GAME	9.3% (41)	8.4% (76)	11.3% (169)
Total	100% (440)	100% (909)	100% (1,498)

During the Introduction Phase, JOURNEY metaphors prevailed in the German media environment, constituting 42.3% of the metaphorical discourse, due to the conceptualization of the newly launched initiative through exploratory and path-oriented metaphorical formulations. During the Expansion Phase, there was an evident escalation in the number of metaphors from the BUILDING type as the government made additional announcements about the launching of various infrastructure initiatives and the first edition of the Belt and Road Forum in 2017. The Contestation Phase ushers in a sharp transition in favor of metaphors from the WAR type, which showed escalation from 18.6 to 31.2% due to intensified geopolitical rivalries, rising doubts within the European community, and the conceptualization of Sino-EU Relations through competition-oriented strategic formulations.

**Figure 3:**
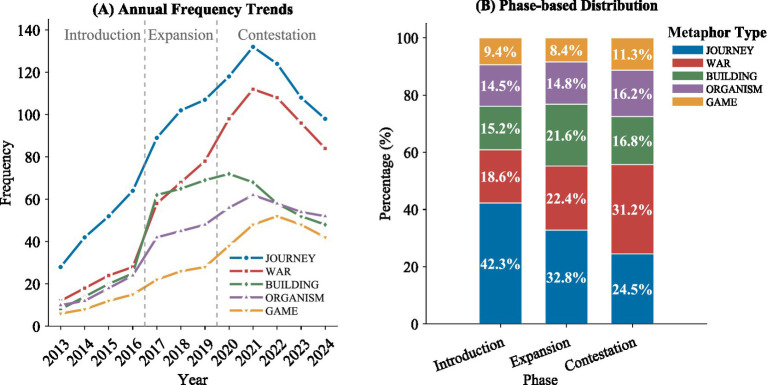
Temporal evolution of metaphorical patterns in German BRI discourse (2013–2024).

As exemplified in Figure 3, the trend line follows the pattern that metaphors of JOURNEY decreased steadily from their peak usage dominance at the beginning of the period when the WAR metaphors showed a steadily increasing trend from the same point in time, especially from the year 2020 onwards. ORGANISM metaphors remained relatively stable throughout the observation period, while there has been moderate growth of the metaphors of GAME from the beginning of the period of the Contestation Phase.

### 4.4 Ideological functions of metaphorical framing

Beyond evaluative orientation, the identified metaphorical patterns perform specific ideological functions in constructing Germany’s geopolitical identity vis-à-vis China and in legitimizing particular policy stances.

Legitimizing European trade defense: WAR metaphors systematically construct the BRI as an economic threat requiring defensive countermeasures. Expressions framing Chinese investment as “invasion,” “conquest,” or “strategic encirclement” naturalize the necessity of protectionist policy instruments such as the EU’s investment screening framework (Comerma, 2025) and anti-subsidy investigations. By mapping economic cooperation onto military aggression, these metaphors foreclose interpretive frames in which BRI projects might be viewed as mutually beneficial.

The making of German geopolitical identity: The interplay between JOURNEY and WAR metaphors reflects Germany’s ambiguous positioning as both China’s economic partner and its geopolitical rival. JOURNEY metaphors, which provide the possibility for shared directionality and mutual benefit, align with Germany’s economic interest in maintaining the relationship with China. Conversely, the prevalence of the WAR metaphors positions Germany in the Atlantic security community as the defender of European sovereignty against the perceived Chinese threat. The temporal shift in the relative importance of JOURNEY and WAR metaphors mirrors Germany’s foreign policy transition from the “Wandel durch Handel” (change through trade) approach to a more security-focused one.

Naturalizing asymmetric agency: In all the metaphor categories, Chinese actors are portrayed as agents (traversing paths, laying foundations, growth/development), whereas European and German actors are portrayed as reactive agents (defending territory, reacting to actions, being influenced by growth). This narrative of asymmetric agency naturalizes a framework in which China acts and Europe merely reacts, thereby obscuring Europe’s proactive engagement in the BRI process.

### 4.5 Summary of key findings

The study of metaphorical framing of the BRI in the German media reveals a number of important findings which throw light on the discourse of the global initiatives of China in the German public sphere. This study has found various trends in the four dimensions of metaphor dominance, the characteristics of the metaphors over time, the differences in the metaphors used across the media platforms, and the differences in the positioning of the metaphors across different national media contexts.

The findings of the analysis show that the JOURNEY metaphors form the preponderant conceptual framework but their relative dominance has fallen dramatically over the period of observation to be offset by the rising dominance of the WAR metaphors. This trend follows the pattern of the securitization of the perception of the Sino-German relationship from being a hoped-for economic partner toward a strategic competitor. The findings of the media outlet analysis reveal notable differences in metaphor preferences across political orientations: the conservative-leaning FAZ shows a markedly higher proportion of WAR metaphors compared to the center-left outlets, which retain a stronger preference for JOURNEY metaphors.

**Figure 4:**
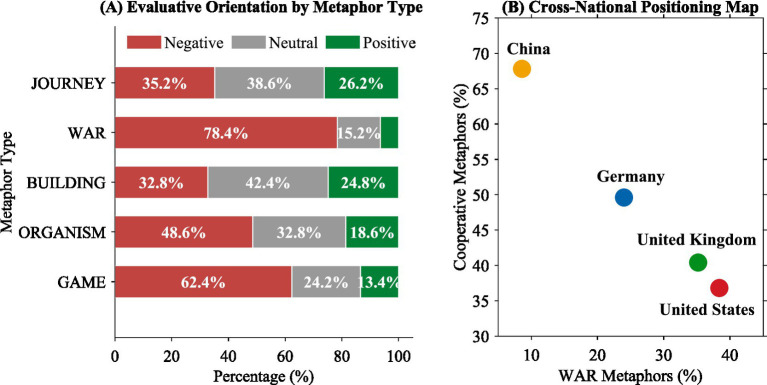
Integrated summary of key findings in German BRI discourse.

As presented in Figure 4, when the findings of this study are considered alongside existing research on BRI discourse in other national contexts (Malik and Hou, 2025; Wei and Hu, 2024; Liu et al., 2023), the German media appear to occupy an intermediary position between the predominantly confrontational framing documented in Anglo-American media and the cooperative narratives characteristic of Chinese state media discourse. The findings of the evaluative orientation analysis reflect that the vast number of metaphorical expressions selected in this study encode negative evaluative pragmas and that the WAR and GAME metaphors are mainly linked to critical assessments of the BRI policy objectives, while the JOURNEY and BUILDING metaphors reflect relatively balanced evaluative profiles. These data support the theoretical hypothesis that metaphorical framing might be a crucial involved mechanism through which the discourse of naturalization of ideological positions takes place.

## 5 Discussion

This study advances scholarship on media discourse surrounding the BRI by revealing the metaphorical mechanisms through which German media shape public perception of this transnational initiative. The predominance of JOURNEY metaphors aligns with broader trends in discourse research on media conceptualization of large-scale infrastructural and geopolitical initiatives, while exhibiting distinct features specific to German media framing.

Placing the present findings in dialogue with existing BRI scholarship reveals both points of convergence and divergence. The identification of JOURNEY metaphors as the dominant frame partially confirms the findings of Wei and Hu (2024), who similarly observed path-oriented conceptualizations across Chinese, Indian, and American news coverage of the BRI, suggesting that this frame may constitute a cross-culturally stable cognitive default for representing large-scale connectivity initiatives. However, the present study challenges the binary opposition implied by prior comparative research, which has tended to contrast confrontational Anglo-American framing with cooperative Chinese framing (Malik and Hou, 2025; Liu et al., 2023). German media discourse, as demonstrated here, occupies a genuinely intermediate position: it reproduces the securitizing logic of WAR metaphors found in Anglo-American outlets while simultaneously retaining a stronger residual presence of JOURNEY and BUILDING metaphors than has been documented in, for instance, US media coverage. Furthermore, the finding that political orientation within a single national context systematically shapes metaphor type preferences extends the conclusions of Kuteleva and Vasiliev (2021), who identified analogous ideological differentiation in Russian media coverage of the BRI, demonstrating that intra-national metaphorical variation is not a Russia-specific phenomenon but a more general feature of BRI media discourse in contexts where the initiative carries genuine political ambiguity.

The dominance of JOURNEY metaphors in the discourse of BRI in the German context has been found to resonate with the findings of research about the Chinese ecological discourse which points to the role of ‘path-oriented conceptualizations’ as the basic cognitive mechanisms of development initiatives (Chunxiang, 2024). This convergence indicates that JOURNEY metaphors have cross-cultural significance when the media create narratives of initiatives based on the concepts of connectivity, progress, and movement in a particular direction. However, this study advances this knowledge by demonstrating that JOURNEY metaphors are analytically productive yet ideologically multidimensional in the context of German BRI discourse, encoding evaluative differences that diverge substantially from the cooperative framing characteristic of Chinese governmental narrative discourse. The study of themes and ideology within the diplomatic discourse of China expresses the fact that the Chinese media employ their narratives of shared development through the usage of cooperation metaphorical phrases (Liu et al., 2023), whereas the metaphors used in the German media’s JOURNEY often convey evaluating aspects regarding the points of destination, the itinerary choices, and the travelers’ real aims.

The increasing prevalence of WAR metaphors reflects a broader discursive shift in Western media coverage of China’s global engagement. Research on US trade policy discourse confirms a parallel trend of repositioning China as a strategic competitor rather than an economic partner (Hu and Li, 2025). While German media discourse mirrors this trajectory, it exhibits distinctive characteristics shaped by the bilateral economic interdependence between Germany and China and Germany’s mediating role within the European framework. The escalation of WAR metaphors from 2020 onwards reflects a confluence of geopolitical triggers, most notably the COVID-19 pandemic, which profoundly reshaped European perceptions of the People’s Republic of China. Scholarship on China’s mask diplomacy of the People’s Republic of China was framed within the first phase of the COVID-19 pandemic has found that the European media framed the story of the Chinese aid in a way that recognized the effort but questioned the strategic motivations behind this effort (Qi et al., 2022), a pattern consistent with the ambivalent metaphorical framing identified in the present analysis.

The cross-national dimension of this research contextualizes German media discourse within the broader global landscape of BRI representations. Drawing on findings from prior studies of BRI coverage in Anglo-American media (Malik and Hou, 2025; Wei and Hu, 2024) and Chinese state media (Liu et al., 2023), the metaphorical patterns identified in this study suggest that German media discourse occupies an intermediary position between the predominantly confrontational framing characteristic of Anglo-American coverage and the cooperative narratives favored in Chinese media. It should be noted that this positioning is inferred from cross-study comparison rather than direct parallel corpus analysis, and future research employing comparable corpora across national contexts would be needed to substantiate this observation empirically. The research study related to strategic narratives of vaccine diplomacy in China illustrates that the media of various nations appropriate and modify the Chinese narratives according to their political context and pre-existing views about China (Stojiljković and Mihelj, 2025), implying the existence of metaphorical framing as an active intersection of the national media culture and geopolitical perspectives rather than a passive reflection of the event itself.

The approach of integrating methodological rigor of corpus-based analysis and critical interpretative insights moves the field of metaphor study forward because it allows researchers to secure deeper insights about the relationship of quantitative metaphor usage patterns and ideological functions. The case study of WAR metaphors in the context of Hong Kong public discourse illustrates the strength of the approach of identification complemented by interpretive insights to identify the political realities constituted through metaphors (Zeng and Ahrens, 2023). The current research expands this methodological framework to apply it to the cross-national comparative study of metaphorical processing of the same target domain in order to systematically study the divergent metaphorical treatment of the target domain across national media regimes.

The differences in metaphorical framing used in the German media of various political inclinations confirm the theoretical expectations concerning the relationship between ideology and metaphorical preferences. Studies concerning the representation of the BRI in the Russian media also point towards the existence of similarities in politically charged metaphorical formulation regarding the media positioning linked to various political inclinations (Kuteleva and Vasiliev, 2021). The current study extends the existing knowledge in the field because it shows that in the same national media context, political ideology systematically shapes the usage of specific metaphor types, as well as the meaning of the implicit assessments carried in rather similar metaphorical statements.

The significance of the above findings can be extended from the discourse analysis method study in academia to real-world applications in the field of intercultural communication and public diplomacy. Studies regarding the usage of frames and the concept of issue linkage in the context of the geoeconomic turn of the EU’s trade policy can convey the ways through which the process of framing discourse has been used as a method of policy formulation in response to the evolving global economic order (Christou and Damro, 2024), implying that the metaphorical building of the BRI through the German media has its implications for the formation of public opinions and eventually policy decisions concerning the engagement of Europe in the Chinese proposal.

Theoretically, this study contributes to illustrating the effectiveness of incorporating Conceptual Metaphor Theory and Critical Metaphor Analysis in the study of politically significant discourse. Although there has been an establishment of the dual role of metaphor as being both cognitive device and ideological tool, there has been limited research integrating the two (Demjén and Semino, 2020). However, the dual role of metaphor has not been systematically investigated through corpus-based research. The framework used in this research allows the replication of a methodological approach which follows the structuring of the mind and the positioning of ideology in metaphorical framing (Semino et al., 2018).

Despite these contributions, this study also recognizes the limitations which qualify the findings and provide guidance regarding the need for subsequent research. The study’s corpus might be said to be representative in its coverage of the large print media in Germany, but it does exclude broadcast media and the discourse of the new media. Additionally, as metaphor identification was conducted by a single coder without independent inter-rater reliability testing, the classification of metaphorical expressions may be subject to individual interpretive bias. Future studies should incorporate double-coding procedures to strengthen analytical reliability.

Future research could scope its metaphorical study to other national contexts in Europe to facilitate a wider comparative approach and the identification of general trends against national specifics. The analytical framework integrating rhetorical analysis with political communication perspectives (Charteris-Black, 2018) could be productively extended to examine how metaphorical patterns in BRI discourse relate to broader argumentative strategies employed in German public debate regarding China policy.

## 6 Conclusion

This research makes a comprehensive critical metaphor analysis of the representation of the Belt and Road Initiative in the German mainstream media from 2013 to 2024. By carefully analyzing 1,247 articles containing a total of 856,432 words, this research found there were 2,847 metaphorical expressions involving the five conceptual frames of the metaphorical expressions. The findings showed JOURNEY metaphors had the highest frequency (29.9%) in the conceptual metaphorical expressions, followed by WAR (26.4%), BUILDING (18.1%), ORGANISM (15.5%), and finally GAME metaphors (10.0%). The findings also showed that there had been a marked transition from the type of cooperation to the type of conflictual representation over time, with WAR metaphors rising from 18.6% during the Introduction Phase to 31.2% in the Contestation Phase. Considered in light of prior research on BRI media discourse in other national contexts, the metaphorical patterns identified in German media suggest an intermediary position between the confrontational framing predominant in Anglo-American coverage and the cooperative framing characteristic of Chinese media. This research aims to promote the understanding of this crucially cognitively structured and ideologically meaningful pattern of the metaphorical framing of the media’s coverage of trans-national initiatives.

## Data Availability

The original contributions presented in the study are included in the article/supplementary material, further inquiries can be directed to the corresponding author.
